# Fully Biobased Polyhydroxyalkanoate/Tannin Films as Multifunctional Materials for Smart Food Packaging Applications

**DOI:** 10.1021/acsami.3c04611

**Published:** 2023-06-02

**Authors:** Martina Ferri, Kseniya Papchenko, Micaela Degli Esposti, Gianluca Tondi, Maria Grazia De Angelis, Davide Morselli, Paola Fabbri

**Affiliations:** † Department of Civil, Chemical, Environmental and Materials Engineering (DICAM), University of Bologna, Via Terracini 28, 40131 Bologna, Italy; ‡ National Interuniversity Consortium of Materials Science and Technology (INSTM), Via Giusti 9, 50121 Firenze, Italy; § Institute for Materials and Processes, School of Engineering, University of Edinburgh, Sanderson Building, Robert Stevenson Road, EH9 3FB Edinburgh, U.K.; ∥ Department of Land, Environment, Agriculture and Forestry (TESAF), University of Padua, Legnaro, Viale dell’Università 16, 35020 Legnaro, Italy

**Keywords:** polyhydroxyalkanoates, tannins, biobased additive, biodegradable polymer, food packaging, smart packaging, active packaging

## Abstract

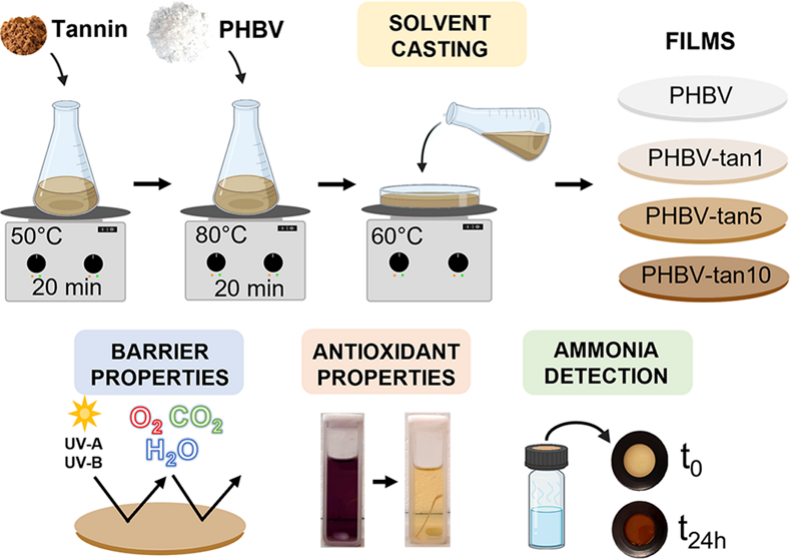

Fully biobased and biodegradable materials have attracted a growing interest in the food packaging sector as they can help to reduce the negative impact of fossil-based plastics on the environment. Moreover, the addition of functionalities to these materials by introducing active molecules has become an essential requirement to create modern packaging able to extend food’s shelf-life while informing the consumer about food quality and freshness. In this study, we present an innovative bioplastic formulation for food packaging based on poly(hydroxybutyrate-*co*-valerate) (PHBV) and tannins as multifunctional additives. As a proof of concept, PHBV/tannin films were prepared by solvent casting, increasing the tannin content from 1 to 10 per hundred of resin (phr). Formic acid was used to reach a homogeneous distribution of the hydrophilic tannins into hydrophobic PHBV, which is remarkably challenging by using other solvents. Thanks to their well-known properties, the effect of tannins on the antioxidant, UV protection, and gas barrier properties of PHBV was evaluated. Samples containing 5 phr bioadditive revealed the best combination of these properties, also maintaining good transparency. Differential scanning calorimetry (DSC) investigations revealed that films are suitable for application from the fridge to potentially high temperatures for food heating (up to 200 °C). Tensile tests have also shown that Young’s modulus (900–1030 MPa) and tensile strength (20 MPa) are comparable with those of the common polymers and biopolymers for packaging. Besides the improvement of the PHBV properties for extending food’s shelf-life, it was also observed that PHBV/tannin could colorimetrically detect ammonia vapors, thus making this material potentially applicable as a smart indicator for food spoilage (e.g., detection of fish degradation). The presented outcomes suggest that tannins can add multifunctional properties to a polymeric material, opening up a new strategy to obtain an attractive alternative to petroleum-based plastics for smart food packaging applications.

## Introduction

In the last decade, the food packaging sector has been experiencing a very quick and deep revolution introducing innovative polymeric materials. The depletion of fossil resources and the large use of fossil-based plastics with short shelf-life have pushed the research to find more and more polymer-based materials starting from renewable resources or valorizing other production wastes.^1^ This is not the only innovation in this field; nowadays, a lot of effort is focused on adding functionalities to the packaging to create the so-called “smart packaging”.^2^ This term includes active packaging, which actively takes part in food conservation,^3,4^ and intelligent packaging, which is able to provide a visual indication of the quality of the contained food.^5^ Typically, active molecules, particles, and/or nanoparticles are used to add the desired functionalities to the polymer that composes the packaging. This leads to smart packaging that is able to protect the foodstuffs from bacterial damage, undesired UV light, and oxygen^6−8^ or to sense and indicate food spoilage.^9^

Several examples of smart packaging, which uses plant-derived substances to enhance polymer properties, have recently been reported. In particular, Athanassiou and co-workers have shown that when poly(lactic acid) (PLA) and curcumin are compounded, the obtained material can colorimetrically detect ammonia vapors.^9^ Moreover, they have developed poly(propylene carbonate)/cinnamon oil fibers that can be used as a delivery platform to control the release of an antioxidant agent only above a certain temperature threshold.^10^ Another recent example of a natural component that can change polymer properties is based on cellulose/naringin blends, which have been presented as multifunctional smart packaging characterized by remarkable UV-blocking properties.^6^

Tannins are polyphenolic extracts that are produced by plants to protect themselves against biotic and abiotic degradation.^11,12^ These plant-based derivatives have been used industrially as raw materials in the leather tannery, and hence they are available in large amounts. From a chemical point of view, defining tannins is particularly difficult due to their heterogeneity in terms of molecular weight and chemical composition. Typically, tannins are classified into hydrolyzable and condensed, and their occurrence depends on the plant source of the extracts.^13^ Hydrolyzable tannins are composed of phenolic groups (such as gallic acids or ellagic acids) esterified with a core sugar structure (generally, glucose). On the other side, condensed tannins are oligomers of flavonoid units that are condensed together. In Europe, most of the commercialized tannin is extracted from chestnut (hydrolyzable tannins), and this product exhibits outstanding antioxidant and antibacterial activities, so it can also be used in the enology and pharmaceutical industry.^13,14^ Moreover, tannins have also found applications as dyes thanks to their intense color, as chemicals for coatings and adhesives, and as additives for food items due to their UV-shielding and antioxidant activities.^13,15,16^

Despite all of the aforementioned properties being also relevant for envisioning the use of tannins in materials for food packaging, their use as a complex mixture has been barely studied for this application. In particular, antioxidant, antibacterial, and UV-blocking properties can significantly inhibit food spoilage. Furthermore, compounding tannins with a biopolymer with suitable high gas barrier properties can result in a fully biobased compound that possesses all properties required for smart packaging in only one material. Among the emerging biodegradable and biobased polymers, polyhydroxyalkanoates (PHAs) represent one of the most promising candidates to replace several conventional fossil-based plastics.^17^ PHAs are well known for their application in the biomedical field as supporting materials for tissue regeneration,^18−20^ and their family consists of more than 150 types of polymers, resulting in broad properties. However, the most studied and simplest member of the group is poly(3-hydroxybutyrate) (PHB), which is similar to polypropylene (PP) in physical properties, but it is highly crystalline and has high melting and glass transition temperatures.^21^ PHB’s application is limited by its narrow processability window, high brittleness, and low impact resistance, among other drawbacks. To improve its properties, 3-hydroxvalerate units can be introduced into the macromolecular chains of PHB to obtain copolymer poly(3-hydroxybutyrate-*co*-3-hydroxyvalerate) (PHBV). Its lower melting and glass transition temperatures, reduced crystallinity, and extended processability window make PHBV more applicable than PHB as a film-forming material for food packaging.^21^ Moreover, several studies have shown that PHBV has good barrier properties toward oxygen and water vapor,^22^ making it a promising material for improving food shelf-life.^23^

Herein, we propose a fully biobased material that combines the properties of both tannins and PHBV with the aim of fabricating transparent multifunctional films for smart food packaging. In particular, different amounts of tannins have been homogenously dispersed in PHBV by a solvent casting method using formic acid as a promising greener alternative to chloroform, which still is the most used solvent to process PHBV due to its very low miscibility in other solvents. This is a further step forward reported by this study, considering the well-known toxicity of chloroform,^24,25^ which makes it definitely unsuitable for food applications even if only small residues remain in the polymeric films. The successful incorporation of tannins gives final flexible polymeric films characterized by antioxidant activity, UV-blocking properties, and high barrier properties toward oxygen and moisture. This study proves that the combination of the properties of these two materials can potentially importantly increase food conservation. Furthermore, it is worth noting that the use of PHBV as the storing material combines the possibility of safely preserving food during its shelf-life, from days for fresh food^26^ to several months for dry food,^27,28^ but also taking advantage of its well-known end-life quick biodegradability.^17^ Moreover, the introduction of biobased and biodegradable tannins^29,30^ is rationally able to maintain the completely high biodegradability of the final material. Further analyses have been performed to investigate the thermal, mechanical, and barrier properties of PHBV compounds and compare them with commercial PLA films, showing properties suitable for the envisioned application in food packaging. Finally, we also noticed that the PHBV/tannin films turn from yellow to dark brown when exposed to ammonia vapor, thus making this material potentially suitable as a smart sensor/indicator embedded in the packaging to have direct visual feedback of the foodstuff quality.

## Results and Discussion

**Scheme 1:**
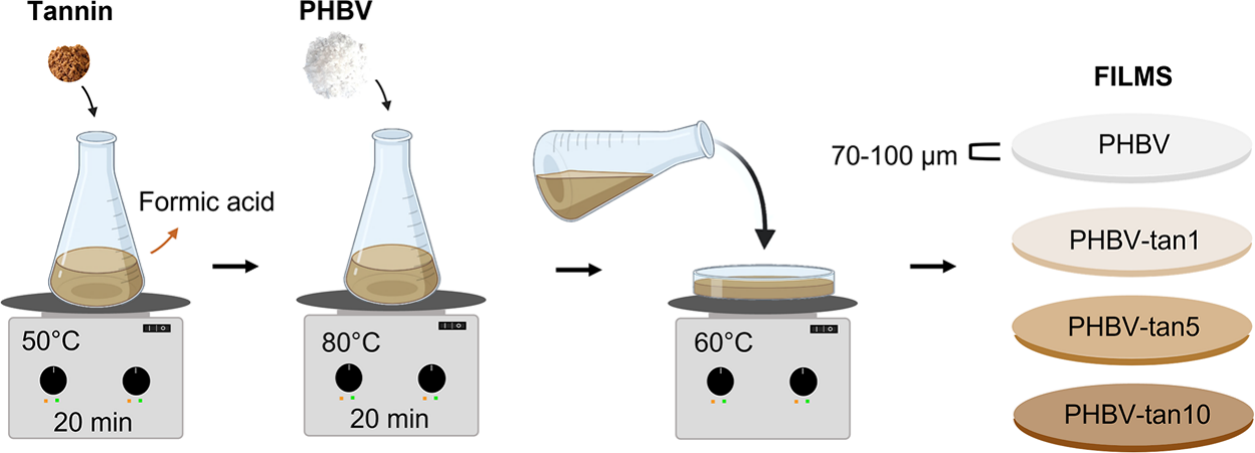
Solvent Casting Conditions and Obtained Films at the Tannin Concentrations of 1, 5, and 10 phr

Nowadays, most food packaging materials are prevalently produced in the shapes of plastic bags, boxes, trays, and flexible films, which are commonly used to preserve fresh food items such as vegetables, fruits, meat, and cheese. As proof of concept, the solvent casting method was employed to prepare neat PHBV and PHBV/tannin films. This preparation procedure requires the selection of a suitable solvent for solubilizing both components. Belonging to the polyhydroxyalkanoates (PHAs) family, it is well known that one of the most commonly used solvents for PHBV is chloroform.^31,32^ However, its extreme toxicity and suspected carcinogenicity make it one of the most hazardous chemicals for the environment and human health.^24,25^ Moreover, considering the high hydrophilicity of tannins, chloroform is not a viable solvent to obtain a homogeneous molecular distribution in a polymeric solution. Recent research has shown that organic acids such as acetic acid and formic acid can be used for the solvent-mediated processing of PHAs.^31,33−35^ After preliminary tests, formic acid was selected as the solvent for film preparation. The films appeared remarkably homogeneous by the naked eye, with good tannin distribution achieved (Figure S1). The three simple steps reported in Scheme 1 summarize the preparation method and the tannin concentrations explored in this research. In particular, PHBV films with 1, 5, and 10 parts per hundred parts of resin (phr) were prepared by dissolving the components in formic acid and drying the obtained solution at 60 °C (the detailed procedure is in Experimental Section).

**Figure 1:**
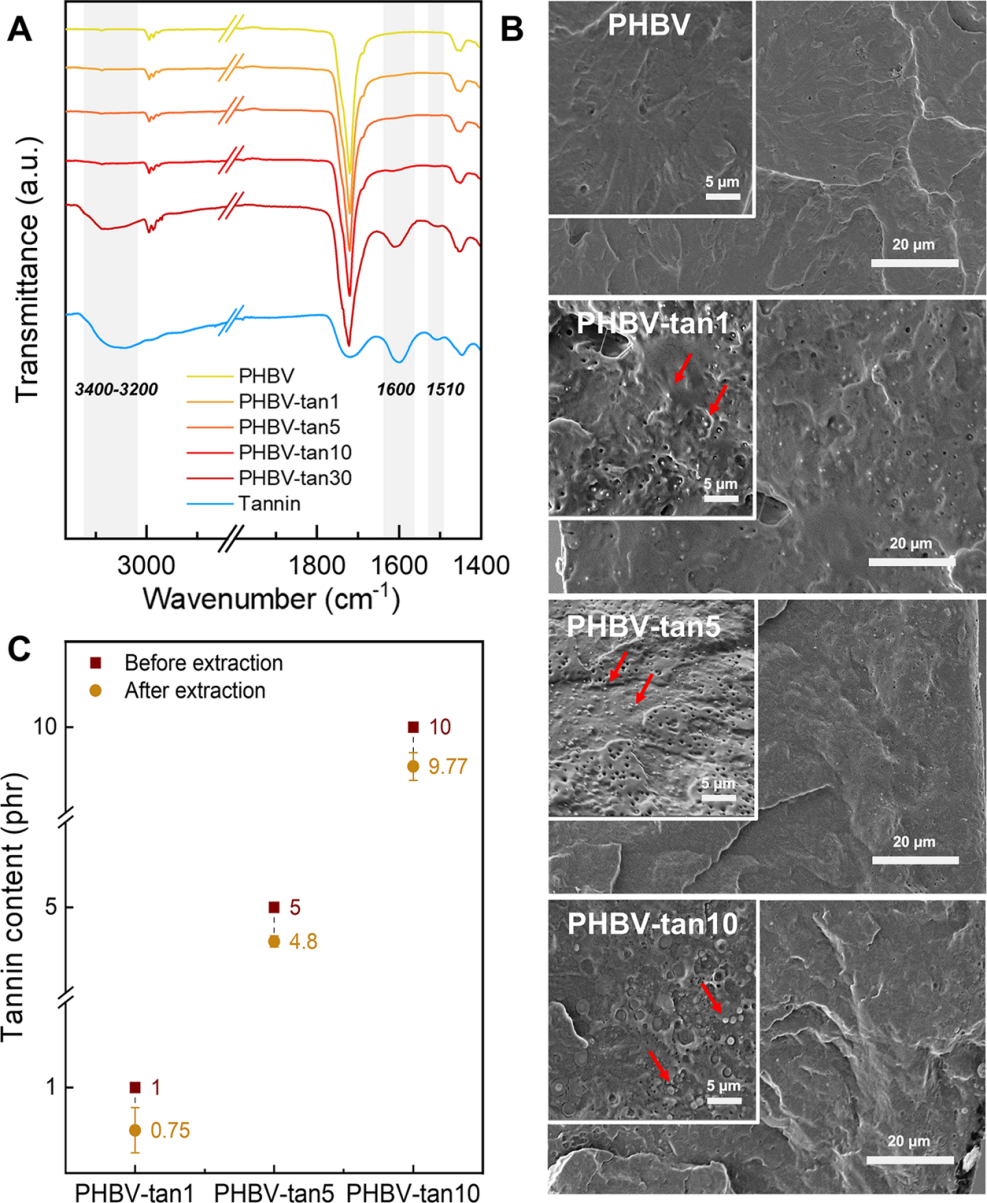
(A) Fourier transform infrared (FT-IR) spectra of PHBV and PHBV/tannin films. (B) Secondary-electron SEM images of PHBV and PHBV/tannin films. The presence of tannin powder is indicated by red arrows. (C) Tannin content (phr) before and after extraction with water. The after-extraction tannin content was calculated by eq 1.

The (ATR) FT-IR analysis was employed to verify the structure of the newly prepared films and understand whether bond formation between the tannins and the polymeric matrix occurred (Figure 1A). Since the tannin concentrations used (up to 10 phr) were too low to obtain sufficient intense bands from the incorporated tannin, a film with 30 phr tannin content was prepared for comparison purposes and analyzed in the same conditions. Chestnut tannins are notoriously classified as hydrolyzable tannins, which belong to the family of gallic or ellagic tannins esterified to an easy sugar.^36^ Their chemical structure is characterized by the band at 1720 cm^–1^, which is associated with the stretching of carboxyl groups.^36,37^ In the same region, the signal of the carbonyl groups of the polyester PHBV is also observed. The addition of tannins does not induce any significant wavenumber shift of this peak, meaning that the transesterification between the matrix and the bioadditive did not occur. However, the increasing intensities of the bands at 3400–3200, 1600, and 1510 cm^–1^, ascribable to O–H stretching, C=C aromatic symmetric stretching, and C=C aromatic asymmetric stretching, respectively, reveal the presence of tannins in the polymeric film.^38^

To better understand the distribution of the bioadditive into the polymer matrix, SEM analyses of cryogenically fractured cross-sectional surfaces of the samples were performed (Figure 1B). At lower magnifications, the film surfaces seem smooth and well represent the typical thermoplastic matrix. At greater magnifications, the tannin powder is clearly visible. Smaller dots are visible in the samples with 1 and 5 phr tannin (average diameters of 0.3 and 0.5 μm, respectively), while bigger aggregates of the additive are formed in PHBV-tan10 (average diameter of 1.3 μm). These images confirm that the incorporation of tannin was achieved at a physical level, in accordance with the FT-IR analysis.

Considering these results, tannin leaching was evaluated by 24 h extraction tests by immersing a specimen in distilled water, which is a highly effective solvent for this compound.^39^ Since neat PHBV resulted in a negligible weight loss of 0.1%, its contribution to the weight loss of PHBV/tannin films was assumed equal to 0. The postextraction tannin content (in phr) was recalculated (by eq 1), as shown in Figure 1C. The new tannin content values remained very close to the nominal ones, suggesting that most of the tannin is retained by the polymer matrix even when forced leaching conditions are imposed. Hence, the observed negligible migration of tannins represented a positive feature of the composite proposed as a potential food packaging material.

**Figure 2:**
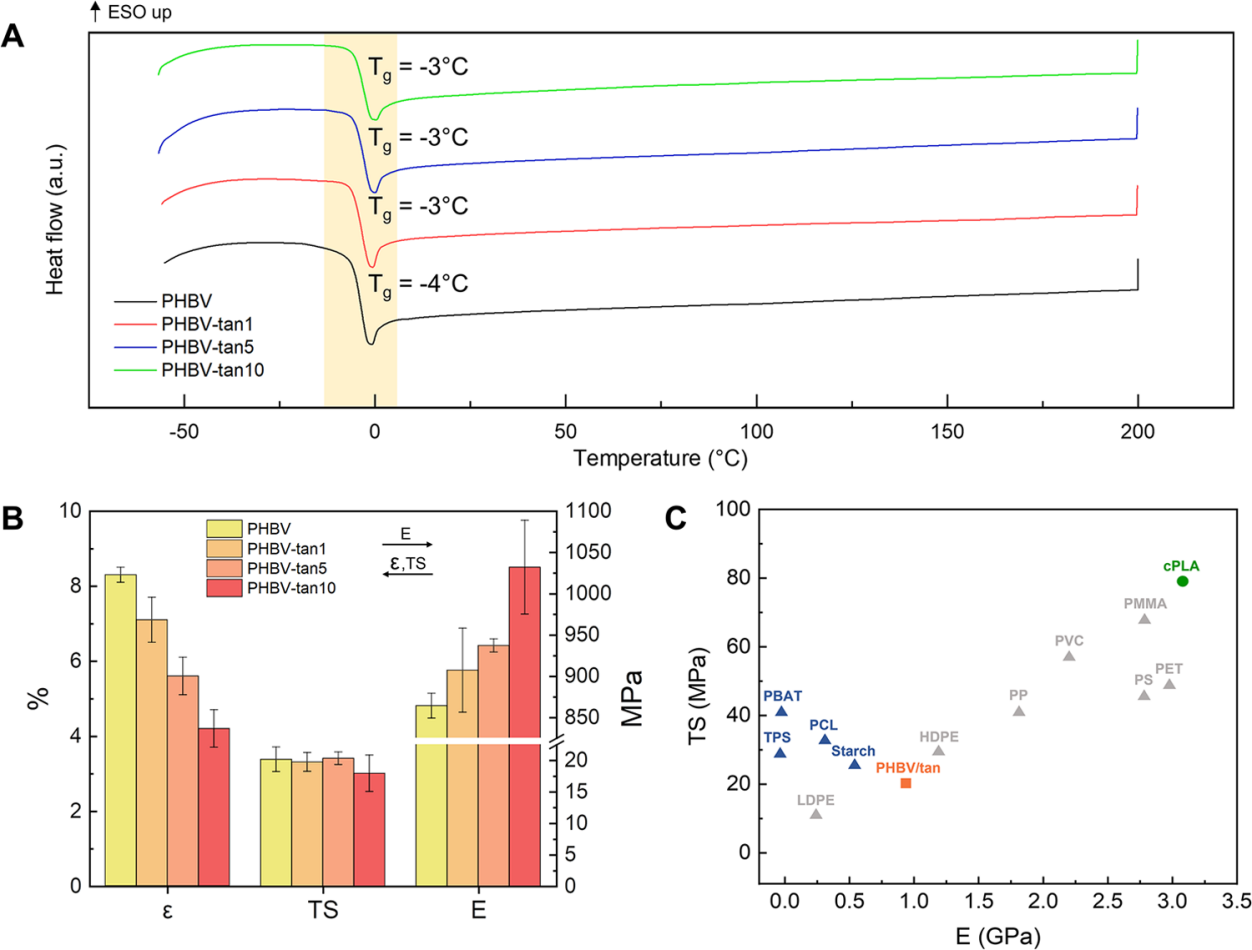
(A) DSC thermograms recorded from the second heating scan of PHBV and PHBV/tannin films. (B) Elongation at break (ε, %), tensile stress (TS, MPa), and Young’s modulus (E, MPa) of PHBV and PHBV/tannin films. (C) Ashby plot of average tensile strength and Young’s modulus data for PHBV/tannin films (orange) compared to the commercial PLA (cPLA, green). The values of Young’s modulus and tensile strength of other common polymers (gray) and biopolymers (blue) were also taken from literature sources and reported as comparison.^6,41,43−45^

Among the requirements of packaging materials for food storage, there is suitability at fridge/freezer temperatures (+4 °C/–18 °C, as suggested by the Food and Drug Administration)^40^ or at high temperatures for microwave or oven heating. Low glass transition temperatures can assure good polymer flexibility at fridge/freezer temperatures; meanwhile, food materials used at high temperatures should have a high melting point.^41^ DSC analyses (thermograms in Figure 2A) were carried out to understand the temperature range where PHBV/tannin films can be employed without thermal transitions that can significantly change their mechanical properties. The results demonstrate that the addition of tannins does not influence the *T*_g_ values of the polymer matrix, which remain nearly stable at about −3 °C. These values mark out the developed material as suitable for storing food in the fridge but probably preclude its application in the freezer, where the temperatures are typically under the *T*_g_ of PHBV, thus importantly reducing its flexibility. Moreover, no thermal transitions are visible between 5 and 200 °C, showing their potential usage also at appreciably high temperatures for food heating.

Another essential function that packaging films must guarantee is the mechanical protection of the stored food to provide a physical barrier during handling and transportation.^41,42^ As displayed in Figure 2B, the incremental addition of tannin content results in a slightly increasing elastic modulus, with a total increment of 20% from neat PHBV to PHBV-tan10. It was previously proved that the addition of polyphenols to a polyester polymeric matrix leads to the formation of hydrogen bonds between the numerous hydroxyl groups of the polyphenolic structure and the ester groups of the polymer, increasing the material stiffness.^36^ Moreover, the introduction of rigid aromatic compounds may hinder the macromolecular movements that could be responsible for the increased stiffness. An opposite trend is shown by the elongation at break, which goes from 8% of neat PHBV to 4% for the 10 phr film. However, the values of tensile stress remain almost stable at around 20 MPa. These results are in line with the morphological aspect already discussed for the SEM images, where the homogeneous tannin distribution and the submicrometric size of the particles slightly increase the stiffness of the material. In general, considering the values obtained in this test, the mechanical properties of all of the PHBV/tannin films are consistent to those of the PHBV previously reported in the literature.^43^ The Ashby plot of tensile stress vs Young’s modulus is presented in Figure 2C to compare the mechanical data of the PHBV/tannin films with commercial packaging based on PLA (cPLA, tested in the same conditions) and with other common plastics and bioplastics from the literature.^6,41,43−45^

Compared to the commercial PLA packaging, the performance of the PHBV/tannin films in terms of stiffness and resistance is reduced. However, the herein-presented material exhibits a tensile strength (TS) value that is located between those of starch and LDPE, and a Young modulus (*E*) value slightly lower than those of starch and HDPE, demonstrating to be potentially comparable to the most used polymers and biopolymers for food packaging applications.

**Figure 3:**
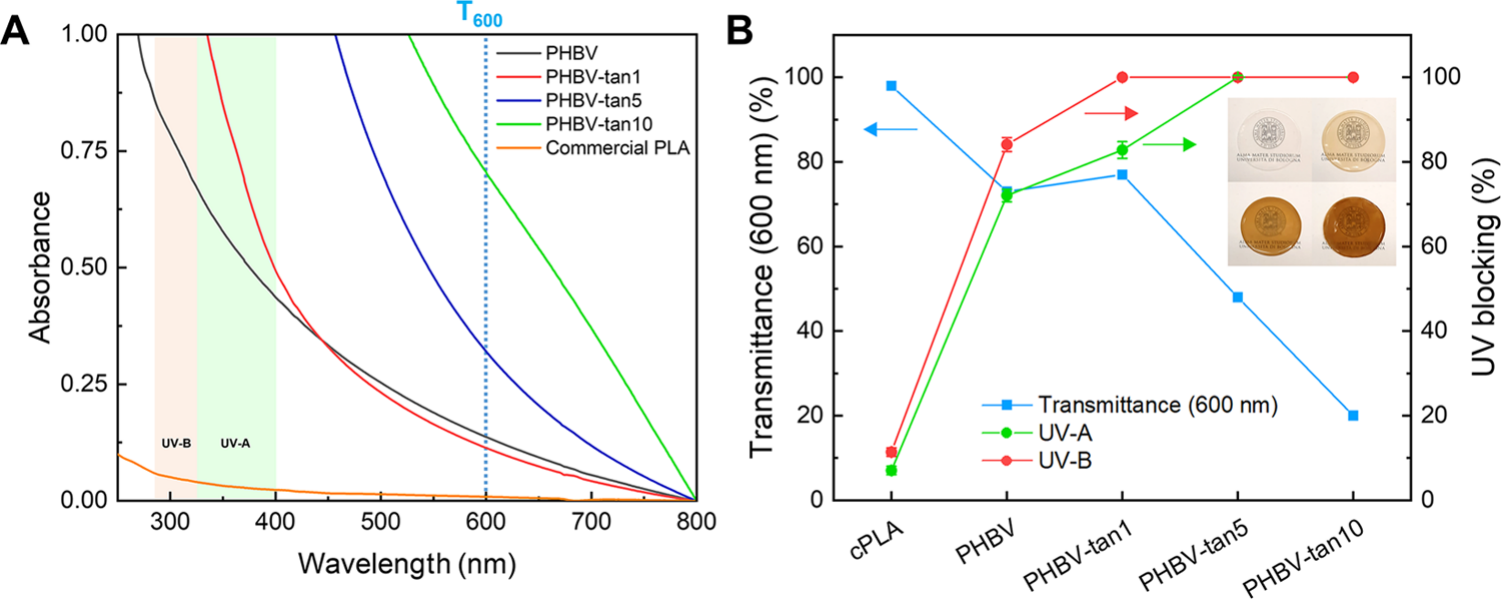
(A) UV–visible spectra of PHBV/tannin films and commercial packaging based on PLA. (B) Transmittance and UV-blocking properties of commercial PLA (cPLA), PHBV, and PHBV/tannin films. UV-A- and UV-B-blocking activity were calculated by eqs 2 and 3, respectively.

From both functional and esthetic points of view, films employed as packaging materials are expected to possess both UV protection and high transparency.^46^Figure 3A presents the UV–visible absorption spectra of PHBV/tannin films compared to commercial PLA-based packaging measured in the same conditions. Despite its almost total transparency (98% at T_600_), the commercial PLA completely lacks UV-blocking activity, as similarly shown by the neat PHBV. On the other hand, UV protection is well exhibited by the PHBV/tannin films. Although 1 phr is insufficient to absorb the whole UV range, PHBV-tan5 and PHBV-tan10 completely block UV-A and UV-B irradiation. This behavior can be attributed to the remarkable UV absorption capacity of the phenolic groups in the tannin molecular structure.^47^ However, adding tannins affects the transparency, which reaches 50% with 5 phr and 20% with 10 phr (Figure 3B). Furthermore, Figure 3B shows the UV-A- and UV-B-blocking activities (calculated by eqs 2 and 3, respectively) in comparison with the transparency variation due to the increasing amount of tannins. Concerning UV protection, it can be inferred that the addition of 5 phr tannins is enough to have a complete blocking of the UV range with an acceptable loss of transparency. Moreover, it is believed that the best compromise between high transparency and UV-blocking activity can be reached by a tannin concentration between 1 and 5 phr. These outcomes confirm the suitability of the developed material for storing food products or photosensitive substances.

A crucial aspect required by a proper packaging material is the gas barrier ability. Reducing the permeability of oxygen is extremely important to guarantee an extension of food shelf-life. Concurrently, suitable permeability to CO_2_ is required when modified atmosphere packages and/or CO_2_-producing foods are involved to mantain a suitable level of CO_2_ in the package. Finally, limiting the exit of water vapor is an effective way to minimize the dehydration of fresh food.^42,48^ In this work, the barrier properties of O_2_, CO_2_, and water were evaluated by direct sorption experiments on PHBV and PHBV-tan5 films.

**Figure 4:**
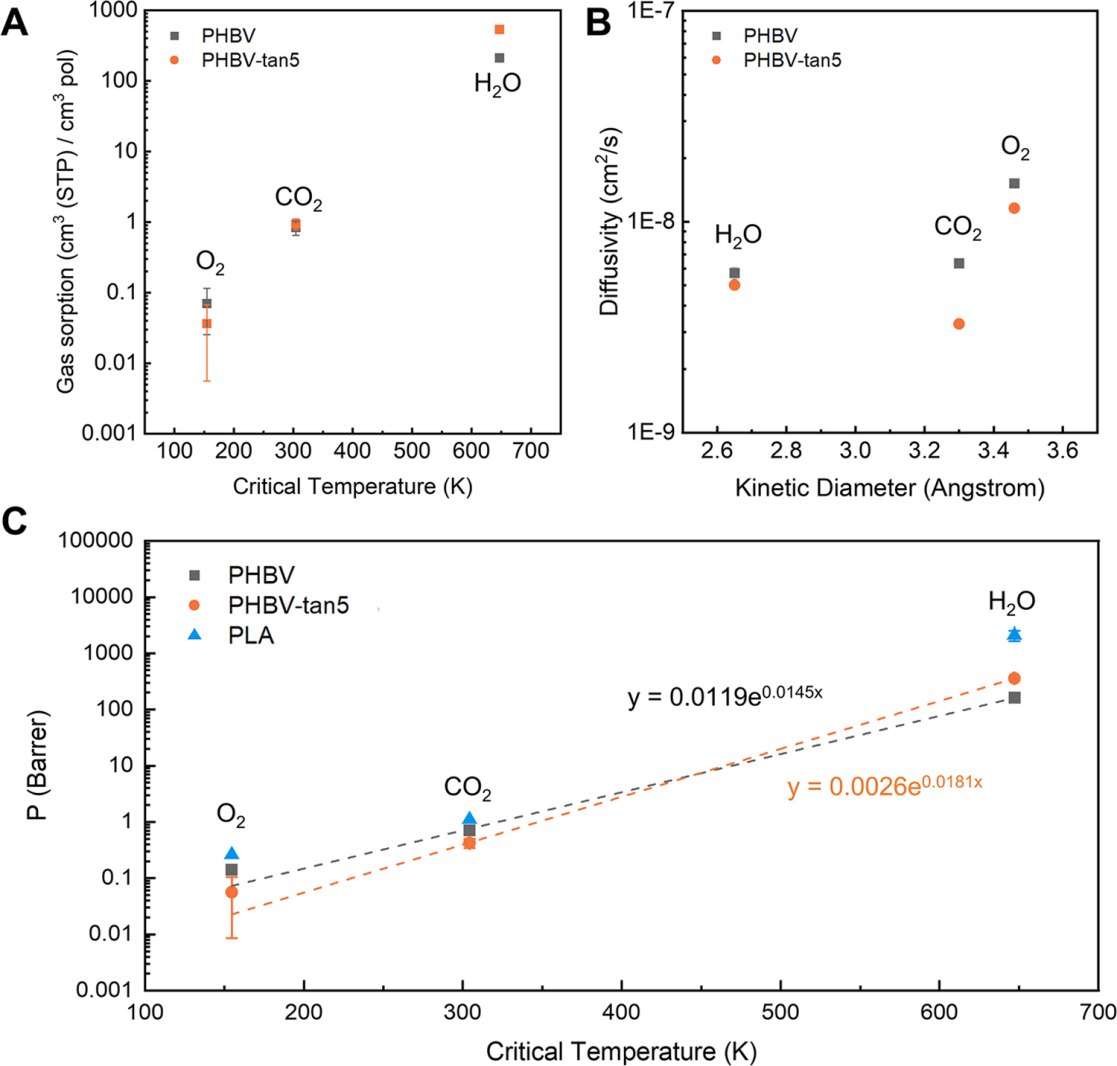
(A) Solubility, (B) diffusivity, and (C) permeability of different penetrants in PHBV and PHBV-tan5. Permeability values for PLA are added for comparison. Permeability values were calculated by eq 4.

Figure 4 shows all of the relevant transport properties in correlation to the properties of pure penetrants, namely, the critical temperature, which gives a measure of the penetrant condensability, and the kinetic diameter, which reflects the gas molecule dimension. Overall, the addition of tannin to the PHBV matrix enhances the solubility of H_2_O and CO_2_, the more polar penetrants, while it lowers the solubility of oxygen (Figure 4A). At the same time, the diffusivity of all penetrants is reduced by the presence of tannins (Figure 4B), which are believed to act as a physical barrier to the path of molecules inside the polymer. The sum of such effects results in the increase of water permeability by a factor of 2.2 in PHBV-tan5 with respect to pure PHBV, while the permeability of CO_2_ and O_2_ decreases by 1.7 and 2.5 times, respectively.

The ideal CO_2_/O_2_ selectivity, defined as the ratio between pure gas permeabilities, can also be an important indicator in food packaging. As the result of tannin addition to the PHBV matrix, the selectivity increased from 5 to 7.4, mostly due to the substantial increase in solubility-selectivity and a slight decrease in diffusivity-selectivity. Indeed, permeability values correlate well with the critical temperature, *T*_c_, of pure penetrants.

Figure 4C shows the permeability values of the three penetrants in PLA, reported in the literature, for comparison.^49−51^ PLA is slightly more permeable to CO_2_ and O_2_ than PHBV and PHBV-tan5, while the CO_2_/O_2_ selectivity is lower than that of PHBV and equal to 4.2. In terms of water permeability, the literature values reported at 25 °C and 100% RH are an order of magnitude higher than those of the materials investigated in this work. Such comparison allows us to conclude that both PHBV and PHBV-tan5 present better barrier performance toward CO_2_, O_2_, and, in particular, moisture uptake than PLA.

The results are also reported in Figure S2 and Table S1 for completeness.

**Figure 5:**
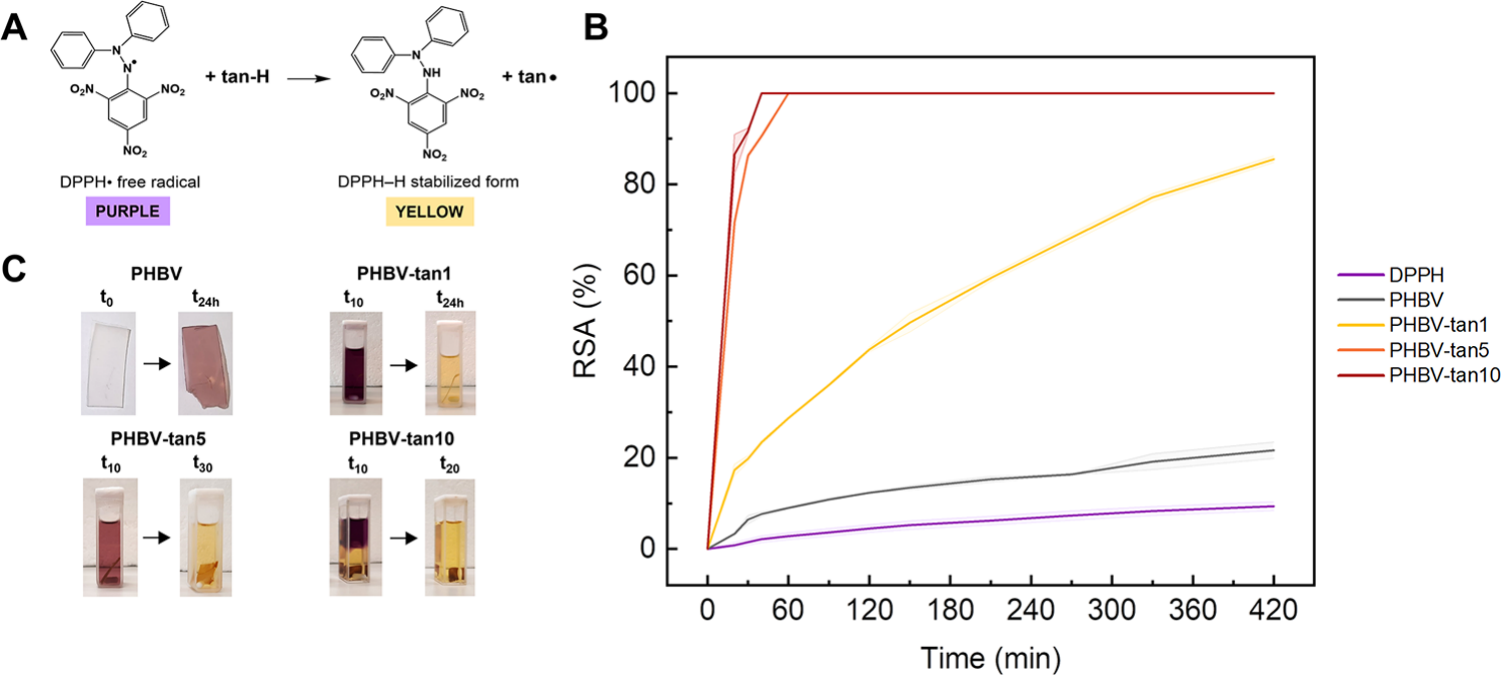
(A) Electron transfer mechanism of DPPH^•^ with the natural antioxidant. (B) Radical scavenging activity (%, calculated by eq 5) of PHBV/tannin films over time. (C) Color transition from white transparent to the pink of the PHBV film after 24 h and color transitions of the cuvettes at different times of analysis.

Although good gas barrier properties are able to protect the stored food from the attack of molecular oxygen, great importance is given to the antioxidant activity that an active packaging material can provide. The evaluation of antioxidant activity of PHBV/tannin films was performed by means of the DPPH^•^ (2,2-diphenyl-1-picrylhydrazyl) assay, which is widely used to simulate the deleterious role of free radicals in food and biological systems. The assay is based on an electron transfer mechanism (Figure 5A), where the DPPH^•^ accepts an electron to become a stable molecule (DPPH–H),^52^ and the newly formed tannin radicals remain stable as a consequence of their numerous resonance forms due to their elevated conjugated and aromatic structure.^38^Figure 5B shows that the radical scavenging activity (RSA%, calculated by eq 5) of the PHBV/tannin films increases with an increase of both the tannin content and time. The slight increase shown by the RSA% of PHBV may be attributed to the physical absorption of the radical DPPH^•^ into the polymer matrix, as demonstrated by the pink color retention of the film after the test (Figure 5C). PHBV-tan1 has a slow but still good RSA, which reaches 80% in 7 h and reaches plateau within 24 h. On the other hand, PHBV-tan5 and PHBV-tan10 exhibit similar and strong antioxidant activity, ensuring the total RSA within the first 20–30 min of analysis. According to the literature,^53,54^ food containing a higher amount of fat (e.g., meat and fish) is well simulated by the ethanol solution employed for the analysis. Taking this into account, the antioxidant activity of the PHBV/tannin films can be correlated with the extension of the shelf-life of fatty foodstuffs because of the limiting of lipid oxidation.

**Figure 6:**
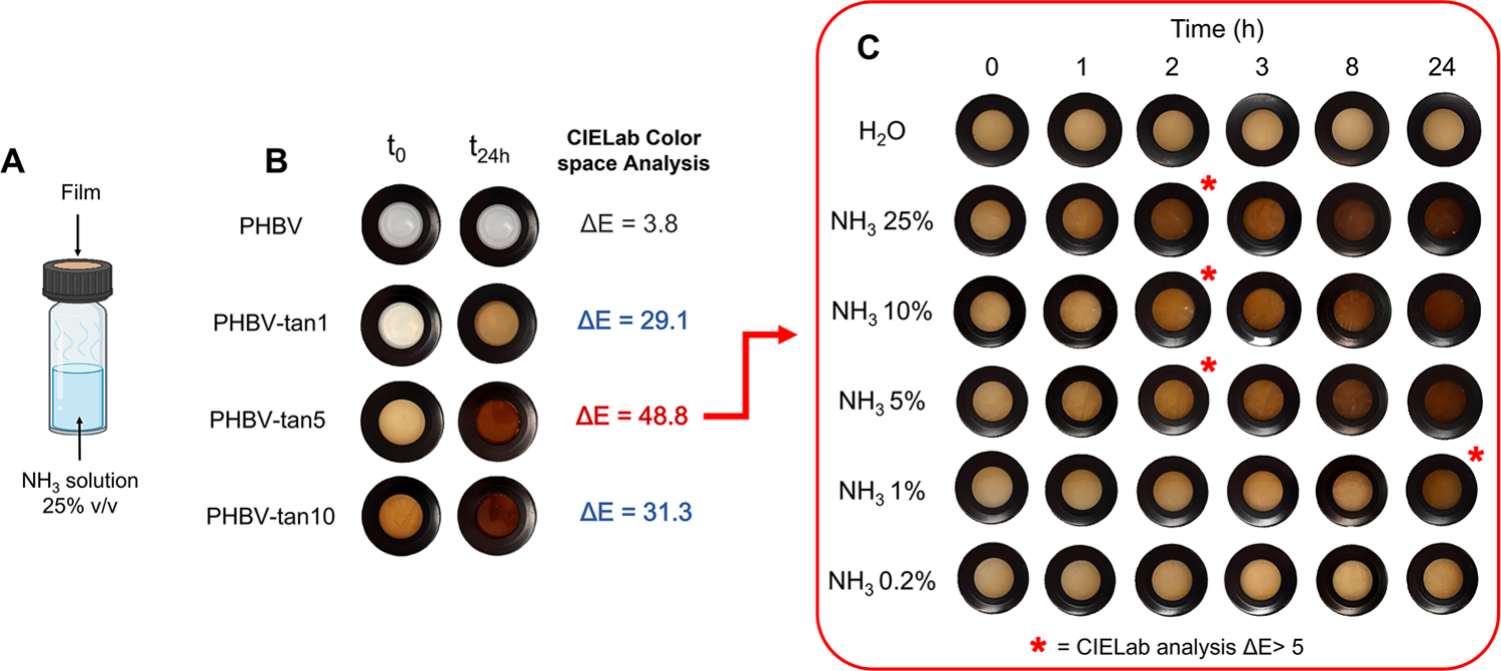
(A) Experimental setup for the ammonia detection test. (B) Color transition of PHBV and PHBV/tannin films after 24 h of exposure to ammonia vapors. (C) Study of the PHBV/tan5 film color evolution over time and with different ammonia solution concentrations.

Spoilage of food is caused by the microbiological activity of various microorganisms, which results in the formation of off-odors and off-flavors that can cause sensory rejection. Among them, biogenic amines and other nitrogen compounds, such as ammonia (NH_3_), trimethylamine (TMA), and dimethylamine (DMA), are associated with the disgusting smell of spoiled protein-rich food, such as meat and fish.^55−57^ Knowing that tannins can form stable complexes with −NH protein groups,^58,59^ the ammonia sensing capability of the PHBV/tannin-based films was investigated by using an aqueous ammonia solution, which easily releases NH_3_ vapors. Figure 6A represents the setup of the test, and Figure 6B shows the color changes of the PHBV/tannin-based films after 24 h exposition. As can be seen, the color transition occurred in all three formulations. This effect can be attributed to acid–base reactions between ammonia gas and tannin structures (hydroxyl, carboxyl, and aromatic groups).^60^ In particular, the darkening phenomenon could be ascribed to the ionization of the hydroxyl groups due to the alkaline environment. The consequent increase of the conjugated system results in a bathochromic shift in the visible spectrum (Figure S3), as already observed by other polyphenolic colorimetric indicators, such as phenolphthalein,^61^ anthocyanin,^62,63^ and curcumin.^64^ The transition was also proved by CIELab color analysis (according to eq 6), which results in Δ*E* > 5 in all of the cases, indicating that the color change is perceivable by the naked eye.^65^ However, PHBV-tan5 exhibited the most significant change (Δ*E* = 48.8, calculated by eq 6) and was selected to test different ammonia solution concentrations (Figure 6C). In general, the color transition was perceivable after 2 h of exposure at up to 5% v/v of NH_3_ solution, and the intensity of the color change from yellow to dark brown increased with the exposure time to NH_3_ vapors. Among the tested concentrations, 1% v/v was the lowest possible to see a significant color transition within 24 h.

## Conclusions

In this work, fully biobased films with multifunctional properties for smart food packaging applications were developed. The addition of multiple functionalities to the biobased and biodegradable matrix poly(hydroxybutyrate-*co*-valerate) (PHBV) was achieved by adding tannins using the solvent casting method, where formic acid effectively worked as a compatibilizing solvent between the hydrophobic polymer and the hydrophilic additive. Fourier transform infrared spectroscopy (FT-IR) and SEM analyses and leaching tests confirmed the homogeneous physical distribution of tannins, which were mostly retained by the polymeric matrix. Thermal analysis (DSC) showed that all of the PHBV/tannin films are suitable for fridge and room temperatures, and the absence of thermal transitions up to 200 °C revealed their potential usage at higher temperatures for food heating. Moreover, Young’s modulus values in the range of 900–1100 MPa and tensile strength at about 20 MPa place the PHBV/tannin material among the most used polymers and biopolymers for food packaging applications. The increase of tannin amounts (1, 5, and 10 phr) progressively lowers the film transparency, but complete UV-blocking activity is achieved when 5 phr tannin is added, suggesting that the best compromise between the two properties lies between 1 and 5 phr. At the same time, PHBV-tan5 and PHBV-tan10 exhibited close and effective antioxidant activity against DPPH^•^ free radicals, proving that 5 phr tannin is enough to give antioxidant power to the developed packaging material. Investigation of the barrier properties revealed that both PHBV and PHBV-tan5 present better barrier performance toward CO_2_, O_2_, and, in particular, moisture uptake with respect to the literature values for PLA. Furthermore, all of the prepared formulations showed a naked-eye visible color change from yellow to dark brown in response to NH_3_ vapor as a food spoilage product model, with PHBV-tan5 having the most relevant change and exhibiting macroscopic significant color transition until 1% v/v NH_3_ solution within 24 h. This newly discovered feature opens the possibility to use them as smart sensors/indicators embedded in the packaging to have direct visual feedback of the foodstuff quality. The herein obtained outcomes suggest that the addition of tannins positively affects the final material’s properties, making it an attractive alternative to traditional plastics for food packaging and representing a promising advancement in smart food packaging technologies and related applications.

## Experimental Section

### Materials

Amorphous poly(3-hydroxybutyrate-*co*-3-hydroxyvalerate) (PHBV, custom grade, *M*_n_ 91,400, *M*_w_ 4,25,700, 25 mol % 3HV, Sigma-Aldrich) was carefully purified by solubilization in chloroform (40 mg·mL^–1^), filtration on celite powder (Standard Super Cel fine, Sigma-Aldrich), and precipitation in a large excess of cold methanol (MeOH, Sigma-Aldrich, ≥99.8%) to completely remove all of the possible bacteria cell residues as detailed described elsewhere.^66^ Chestnut (*Castanea* spp.) tannins (Saviotan, kindly supplied by Saviolife) were used as received. The thermal stability of the used tannins was determined by thermogravimetric analysis (TGA), as described in the Supporting Information. Figure S4 presents the TGA and DTGA curves of the tannins. Formic acid (HCOOH, ≥98%) and ethanol (EtOH, ≥99.8%) were purchased from Sigma-Aldrich and used without further purification.

### Samples’ Preparation

PHBV/tannins thin films (thickness of approx. 80 μm) were prepared by the solvent casting method. Depending on the final tannin concentration in the film (1, 5, 10 phr), the proper amount of tannin powder was first solubilized in 13 mL of formic acid under magnetic stirring (50 °C, 20 min). Then, PHBV was added (700 mg), and the mixture was stirred at 80 °C until the complete solubilization of the biopolymer. Next, the solution was poured into a glass Petri dish (diameter 11 cm) and dried at 60 °C in a fume hood using a covering box to protect the film from the airflow. The prepared film samples were stored at room temperature for 24 h prior to characterization.

### Characterizations

#### Fourier Transform Infrared (FT-IR) Spectroscopy

FT-IR was conducted using a PerkinElmer Spectrum Two spectrometer equipped with a diamond crystal in attenuated total reflectance (ATR) mode. Spectra were recorded in the wavenumber region between 4000 and 400 cm^–1^ across 16 scans using a spectral resolution of 4 cm^–1^. Spectral data were processed with Spectrum 10 software (PerkinElmer).

#### Scanning Electron Microscopy (SEM)

The cross-sectional microstructures of the samples were investigated using a Nova NanoSEM 450 electron microscope (FEI Company). Prior to analysis, the samples were subjected to cryogenic fracture using liquid nitrogen (N_2_) and placed onto a copper tape sicked to an aluminum pin stub. A thin layer of gold (approx. 10 nm) was deposited on the surface of the samples by the electron deposition method to avoid the surface charging effect. The analysis was conducted by applying an accelerating voltage of 5 kV.

#### Extraction in Water

One piece (40 mm × 20 mm) of each film was weighed (*w*_1_) and then immersed in 50 mL of distilled water for 24 h under low-stirring conditions (100 rpm), ensuring no collision between the magnet and the sample. Next, the films were dried for 3 h at 75 °C under dynamic vacuum. Then, the dried samples were kept in a fume hood for 1 h before weighing (*w*_2_). The values of the tannin contents (in phr) after the extraction were calculated as follows
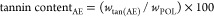
1where *w*_tan(AE)_ is the tannin content in the weighted piece after the extraction and *w*_POL_ is the weight of the only polymer (without tannin) in the analyzed specimen. These were calculated as follows
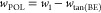

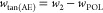
where *w*_tan(BE)_ is the tannin content in the weighted piece before the extraction and is obtained as follows
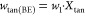
where *X*_tan_ is the nominal tannin fraction in the analyzed specimen. The value of *w*_tan(AE)_ was calculated assuming that the neat PHBV (tested as a reference) does not lose any weight during the extraction analysis. All of the results were given as mean values and standard deviations from at least two measurements.

#### Differential Scanning Calorimetry (DSC)

Thermal phase transitions of the prepared films were investigated by differential scanning calorimetry (Q10, TA Instruments), fitted with a standard DSC cell and equipped with a Discovery Refrigerated Cooling System (RCS90, TA Instruments). The system was calibrated both in temperature and enthalpy with an indium standard. A ramp at a heating rate of 20 °C·min^–1^ from 25 to 200 °C was performed before taking measurements to eliminate any trace of water. The samples (3 mg) were placed into aluminum pans and subjected to two heating cycles from −60 to 200 °C at a heating/cooling rate of 10 °C·min^–1^ under a nitrogen purge of 20 mL·min^–1^. Isothermal steps of 1 min were employed to equilibrate the samples at the interval boundary temperatures. After quenching the thermal history of the samples through the first heating cycle, the curves from the second heating scan were processed with TA Universal Analysis 2000 software (TA Instrument) to extrapolate the glass transition temperature (*T*_g_).

#### Mechanical Properties

The tensile properties of the PHBV/tannin films were assessed on an Instron 5966 device (Instron), equipped with a 10 kN load cell. Specimens in the form of stripes (length 70 mm, width 10 mm) were cut from the films and tested at a crosshead speed of 10 mm·min^–1^. The tensile properties were measured depending on the film thickness, which was calculated for each sample by using the arithmetic mean of a threefold determination. The results were reported as mean values and standard deviations from at least five measurements.

#### Optical Properties

UV–vis spectroscopy was used to evaluate the optical properties of PHBV/tannin films. With a thickness of approx. 50 μm to match that of common packaging films, spectra within the range of 200–800 nm at a resolution of 0.5 nm were collected for each formulation on a Jasco V-650 spectrophotometer operating in the absorbance mode. The reported spectra were obtained by normalizing the absorbance values according to the thickness of each film. The UV-blocking activity was calculated by using the following equation
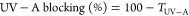
2
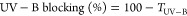
3where *T*_UV-A_ and *T*_UV-B_ are the average transmittance values in the corresponding spectral regions (UV-A from 400 to 315 nm; UV-B from 315 to 280 nm). Three measurements per sample were performed. The outcomes were averaged, and standard deviations were determined.

#### Barrier Properties

CO_2_ and O_2_ transport properties were determined at 30 °C by direct sorption in a manometric closed volume, variable-pressure (pressure-decay) apparatus following ASTM D1434. A known amount of gas was fed into the sample chamber, and the mass uptake was evaluated by measuring the pressure decrease in the gaseous phase over time. The solubility coefficient (*S*) was then extracted as the ratio between the equilibrium concentration of gas in the polymer and the corresponding pressure, while the diffusion coefficient (*D*) was evaluated from sorption kinetics by considering Fickian diffusion and the variation of interfacial concentration during the experiments, as described elsewhere.^67−69^

Water transport properties were evaluated at 30 °C and 100% RH through direct gravimetric measurements. Samples were immersed in deionized water for a specific amount of time, periodically removed, carefully dried, and weighed immediately, until full saturation, according to ASTM D570.^70^ Moisture uptake in time, *m*_w_ (*t*), was calculated by using the following equation
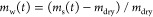
where *m*_s_(*t*) is the mass of the sample at time *t* and *m*_dry_ is the initial mass of the sample. All specimens were conditioned at 50 °C prior to testing to remove humidity absorbed from the atmosphere to an equal amount. An example of the sorption curve is reported in Figure S2.

The solubility coefficient (*S*) of water was then calculated as the ratio between the equilibrium concentration of water in the polymer and the saturation pressure of water at the given temperature. Sorption kinetics was used to evaluate the diffusion coefficient (*D*) by considering Fickian diffusion.^69^ Water flux can be evaluated by using the following equation
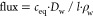
where *c*_eq_ is the equilibrium concentration, *l* is the film thickness, and ρ_w_ is the water density at the temperature of the test.

For all penetrants, the permeability was evaluated under the assumption of the validity of the solution-diffusion model,^71^ as follows
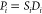
4

The ideal selectivity, useful for evaluating the film performance, is defined as follows
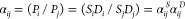
where α_*ij*_^*S*^ and α_*ij*_^*D*^ are the solubility and diffusivity contributions to the selectivity, respectively.

The results are reported as mean values and standard deviations from at least two measurements.

#### Antioxidant Activity

**Scheme 2:**
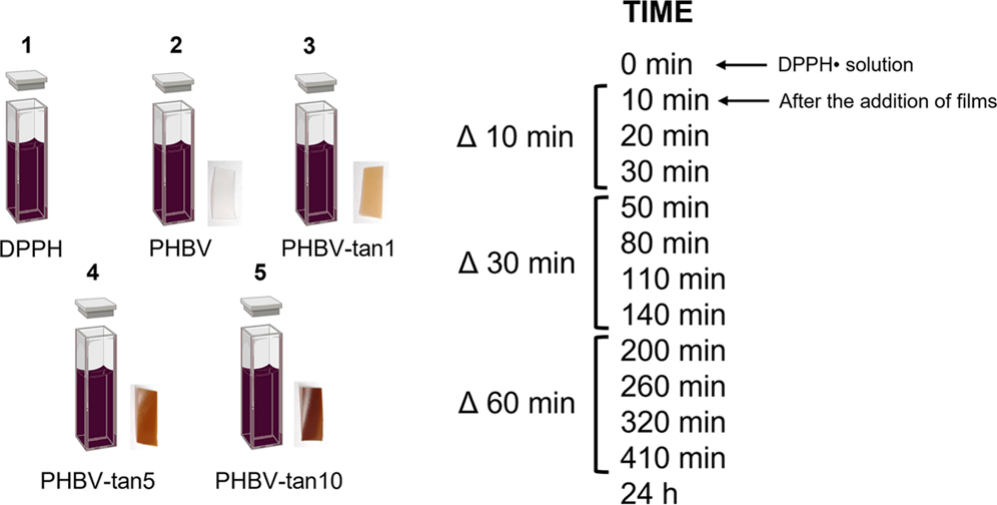
Schematic Overview of the Measurements to Evaluate the Antioxidant Activity

The antioxidant activity of the PHBV/tannin films was investigated by the DPPH^•^ free radical scavenging method.^72^ First, a 0.2 mM solution of DPPH^•^ in ethanol was prepared and poured into five cuvettes. The experiment started by measuring the absorption spectrum of each cuvette containing the DPPH^•^ solution (*t*_0_). Then, immediately after the acquisition of the last spectrum, small pieces (0.5 cm × 1.5 cm) of the PHBV/tannin films (thickness ≈ 240 μm) were put into four out of the five cuvettes. Since light and oxygen can affect the absorbance of DPPH^•^,^73^ the fifth cuvette was left as the DPPH^•^ reference, monitoring its self-decay. The spectrum of each cuvette was recorded by a Jasco V-650 spectrophotometer in the range of 700–200 nm (resolution of 0.5 nm) at specific times, as shown in Scheme 2.

The antioxidant activity was calculated according to the following equation
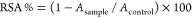
5where *A*_sample_ is the maximum absorbance at the specific measurement time and *A*_control_ is the maximum absorbance of each cuvette at *t*_0_. All results are reported as the mean values for different samples from at least two experiments.

#### NH_3_ Detection

The assessment of the PHBV/tannin film’s capability to detect ammonia vapors was investigated by performing two tests. For both, circular-shaped samples with a diameter of 18 mm and a thickness of 100 μm were cut from the PHBV/tannin films and integrated within the holed caps of vials containing aqueous ammonia solution (≈3 mL). The vials were left under the hood for a total of 24 h. In the first test, 25% v/v ammonia solution was used and all formulations were examined. In the second test, PHBV-tan5 was kept constant while changing the NH_3_ concentration (25, 10, 5, 1, 0.2% v/v). Both experiments were conducted under laboratory conditions.

The quantification of the color transition of the films was performed by using the CIELab color space system, an analytical tool that defines colors through a unique combination of Cartesian coordinates (L*, a*, and b*). Photos of the samples were taken under the same distance and light. Then, the values of L*, a*, and b* were captured by using free mobile application “deltacolor”, which gives the total color difference Δ*E*, according to the following equation
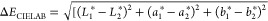
6

When the value of Δ*E* exceeds 5, two different colors are clearly perceived by the observer.^65^

## Supporting Information Available

Sample photographs, water uptake curve in the water sorption experiment, detailed data table of the gas barrier properties, UV–visible spectra before and after ammonia fuming, thermogravimetric analysis experimental details of chestnut tannins, and related TGA and DTGA curves (PDF)
