# Supplementary material for: Fully Biobased Polyhydroxyalkanoate/Tannin Films as Multifunctional Materials for Smart Food Packaging Applications
Source: ACS Appl Mater Interfaces. 2023 Jun 2;15(23):28594–605. doi: 10.1021/acsami.3c04611 (PMC10273181; doi:10.1021/acsami.3c04611)
Supplement: Supplementary file 1 — am3c04611_si_001.pdf [file am3c04611_si_001.pdf]

SUPPORTING INFORMATION

Fully bio-based polyhydroxyalkanoates/tannin films as multifunctional materials for smart food packaging applications

Martina Ferri [a,b], Kseniya Papchenko [c], Micaela Degli Esposti [a,b,*], Gianluca Tondi [d], Maria Grazia De Angelis [c], Davide Morselli [a,b,*], Paola Fabbri [a,b]

[a] *Department of Civil, Chemical, Environmental and Materials Engineering (DICAM), Università di Bologna, Via Terracini 28, 40131 Bologna, Italy*

[b] *National Interuniversity Consortium of Materials Science and Technology (INSTM), Via Giusti 9, 50121 Firenze, Italy*

[c] *Institute for Materials and Processes, School of Engineering, University of Edinburgh, Sanderson Building, Robert Stevenson Road, Edinburgh EH9 3FB, UK*

[d] *Department of Land, Environment, Agriculture and Forestry (TESAF), Università di Padova, Legnaro, Viale dell'Università 16, 35020 Legnaro, Italy*

* Corresponding Author

Davide Morselli
office: +39 051 2090363
davide.morselli6@unibo.it

Micaela Degli Esposti
office: +39 051 2090363
micaela.degliesposti@unibo.it

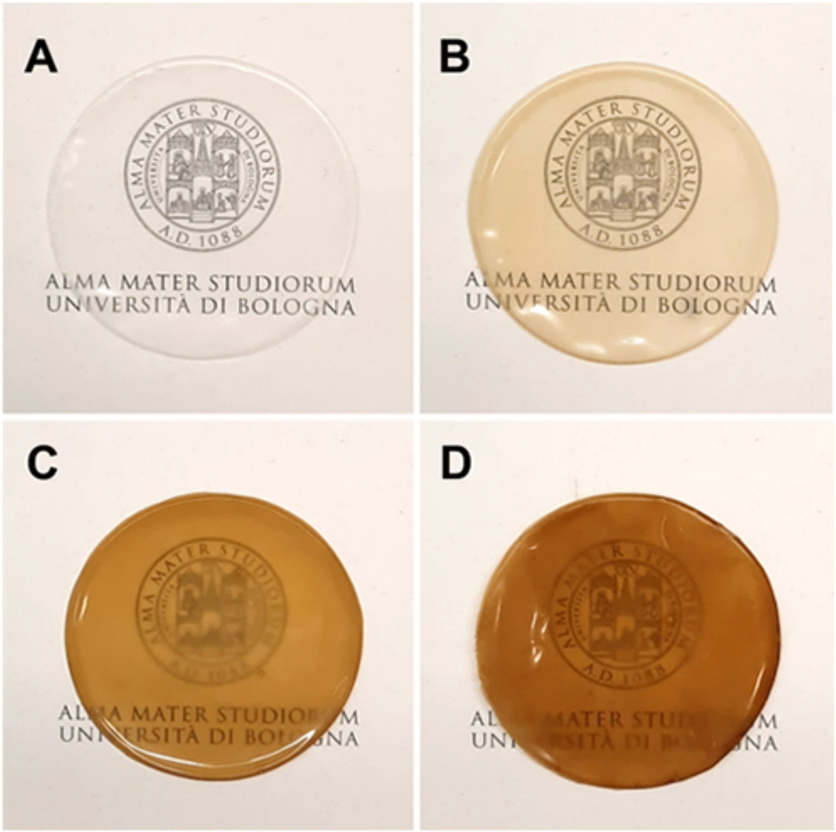

Figure S1. (A) PHBV and PHBV/tannin films at (B) 1 phr, (C) 5 phr, and (D) 10 phr.

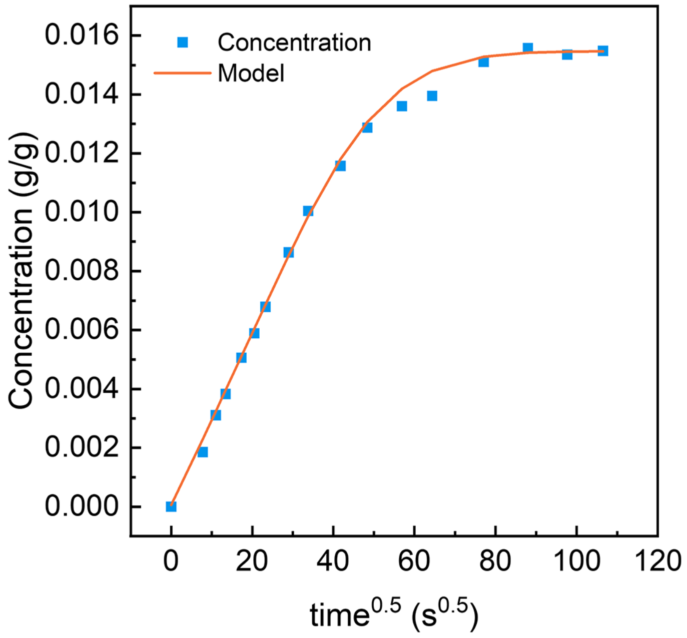

Figure S2. Example of water uptake curve during the water sorption experiment (PHBV-tan5, thickness 85 µm).

Table S1. Data used for correlation. Gas sorption experiments were conducted at equilibrium pressure of 1.5 bar. The reported PLA values are taken from the literature.[1–3]

			PHBV			PHBV-tan5			PLA
	Kin diameter (Angstrom)	*Tc (K)*	*S (cm^3/cm^3 bar)*	*D* $\cdot 10^{-9}$ *(cm^2/s)*	*P (Barrer)*	*S (cm^3/cm^3 bar)*	*D* $\cdot 10^{-9}$ *(cm^2/s)*	*P (Barrer)*	*P (Barrer)*
**H_2O**	2.65	647.1	211.8 ± 19.3	5.7 ± 0.4	161.1 ± 6.0	535.9 ± 12.9	5.0 ± 0.3	358.3 ± 24.7	2078 ± 449
**CO_2**	3.3	304.25	0.84 ± 0.19	6.4 ± 0.2	0.71 ± 0.16	0.96 ± 0.17	3.3 ± 0.1	0.42 ± 0.08	1.1
**O_2**	3.46	154.6	0.07 ± 0.04	15.2 ± 0.5	0.14 ± 0.09	0.04 ± 0.03	11.6 ± 0.4	0.06 ± 0.05	0.26

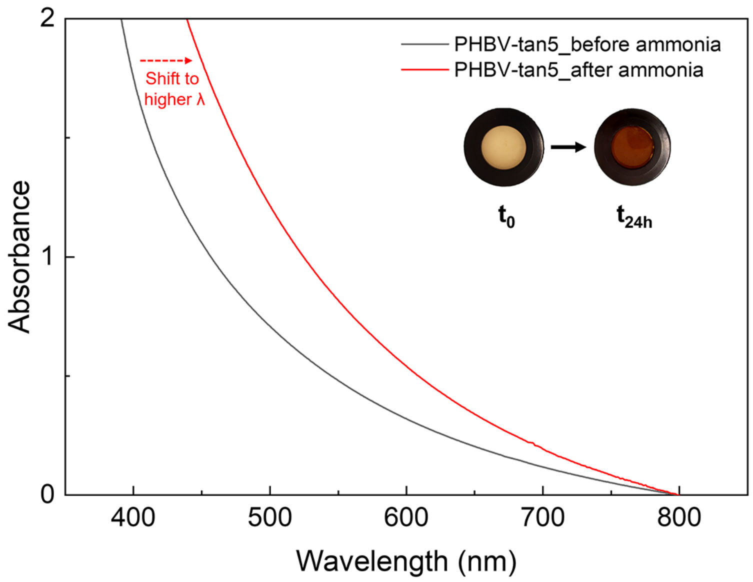

Figure S3. Absorbance UV-vis spectra of PHBV-tan5 before and after 24 h of exposition to ammonia vapors.

Thermogravimetric analysis experimental details

The thermal stability of chestnut tannins was investigated by thermogravimetric analysis (TGA, TGA4000, PerkinElmer) on a sample of about 15 mg, placed in a ceramic pan, through a temperature ramp of 10°C·min^{-1} from room temperature up to 900°C. The test was carried out in an inert environment by using a nitrogen flow. The weight loss (%) of the sample as a function of temperature was recorded by using Pyris software. The first derivative (DTGA) was then calculated to highlight the degradation peaks.

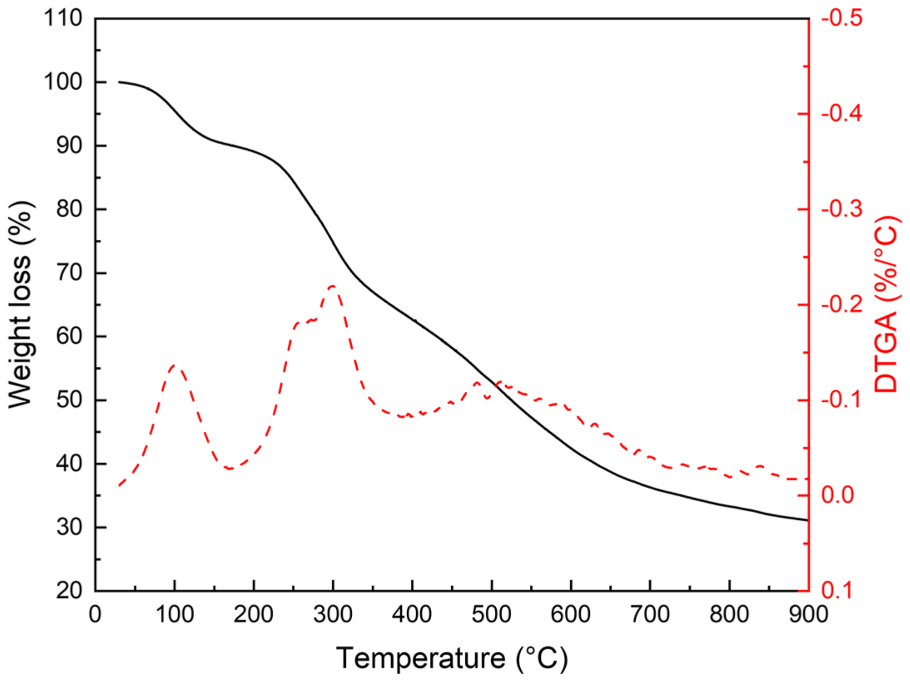

Figure S4. TGA (black line) and DTGA (red line) thermograms of chestnut tannins.

References

(1) Siparsky, G. L.; Voorhees, K. J.; Dorgan, J. R.; Schilling, K. Water Transport in Polylactic Acid (PLA), PLA/ Polycaprolactone Copolymers, and PLA/Polyethylene Glycol Blends. *J Environ Polym Degrad* **1997**, *5* (3), 125–136. https://doi.org/10.1007/BF02763656.

(2) Auras, R. A.; Singh, S. P.; Singh, J. J. Evaluation of Oriented Poly(Lactide) Polymers vs. Existing PET and Oriented PS for Fresh Food Service Containers. *Packaging Technology and Science* **2005**, *18* (4), 207–216. https://doi.org/10.1002/pts.692.

(3) Fabra, M. J.; Lopez-Rubio, A.; Lagaron, J. M. Nanostructured Interlayers of Zein to Improve the Barrier Properties of High Barrier Polyhydroxyalkanoates and Other Polyesters. *J Food Eng* **2014**, *127*, 1–9. https://doi.org/10.1016/j.jfoodeng.2013.11.022.
